# ‘Why can’t you just be fine?’: An autoethnography of self-harm from a lived experience and nursing perspective

**DOI:** 10.1177/13634593251342902

**Published:** 2025-05-26

**Authors:** Caroline da Cunha Lewin

**Affiliations:** King’s College London, UK

**Keywords:** anorexia nervosa, autoethnography, qualitative, self-harm, survivor

## Abstract

Psychiatric discourse problematises self-harm as a psychopathological behaviour indicative of individualistic deficiency. This guides clinical priorities in treatment whilst negating salient components and individual preferences. Conversely, survivor-controlled research emphasises underacknowledged aspects of self-harm, such as its embodied emotionality as embedded within sociocultural context. This suggests a need for re-theorisation. Autoethnography (AE) utilises the researcher as the main source of data to elucidate social phenomena. Through AE, I consider my lived and professional experiences, as a registered general nurse, of self-harm by referring to my medical notes, memory reflections and personal diary entries as contextualised to self-harm literature. This lived experience (LE) perspective of self-harm is derived from subjective experience and contemporary literature, framed within survivor epistemology. This novel understanding argues that people with self-harm may experience immersive, aversive embodied emotionality arising from sociocultural and relational conflict. It considers self-harm as supporting the person to (1) be an integrated whole; (2) employ self-care; and (3) connect with oneself and others. This LE perspective directly critiques dominant psychiatric conceptualisations, instead compassionately framing self-harm as socially implicated. This could improve societal understanding, reduce pejorative attitudes and benefit people with LE.

## Introduction

Psychiatric discourse conceptualises self-harm as any self-injurious behaviour that causes physical harm to one’s body. This conceptualisation is framed within the biomedical approach as a destructive symptom of underlying mental ‘illness’, and indicative of emotional dysregulation (Chandler et al., 2011). However, there are discrepancies as to the conceptualisation of its methods and intention (Chandler et al., 2011). For instance, in the US, the definition is narrower, and suicidality is excluded, often referring to non-suicidal self-harm (NSSI), whereas in the UK, motivation is considered irrelevant (Chandler et al., 2011; Klonsky et al., 2014). Moreover, whilst self-cutting is most widely recognised, some acknowledge eating distress and substance misuse as self-harm (Farber Klayman, 2002). However, these are also emphasised as diagnosable entities (Farber Klayman, 2002).

In conceptualising self-harm as psychopathological, clinical research seeks to develop individualised treatment-oriented solutions, such as pharmacological, restrictive or preventative interventions, to achieve a ‘cure’ (Beresford et al., 2010). This compounds moral and legal responsibilities in healthcare, ‘to keep someone safe and “fix” things’ (Baker et al., 2013: 33). Environmental and personal factors, such as childhood abuse, trauma, female gender and identifying as LGBTQIA+, are also identified as salient (Chandler et al., 2011). However, these are referred to tokenistically, without exploring how these experiences may be inherently interrelated or how they are subjectively experienced (Gurung, 2018). Overall, in psychiatric discourse, self-harm is problematised, failing to consider LE perspectives and excluding people’s needs, which may differ significantly from clinical priorities (Chandler, 2016).

Alternative theorisations construct self-harm as a far more than a behaviour. For instance, psychoanalytic theory may perceive self-harm as a re-imagining of relational distress, whereby there is conflict between an internalised ‘abuser’, as enacting the self-violence, and the person, who cares for themselves (Motz, 2009; Turp, 2003). Feminism may similarly acknowledge self-harm within sociocultural context, perceiving it as internalised patriarchal discourse, a means to simultaneously respond to and maintain, gendered expectations (Malson, 1998; McAndrew and Warne, 2005). Phenomenology emphasises the embodied meaning and felt experience of self-harm in connection with, and experienced by, others (Brown and Kimball, 2013; Heney, 2020). Survivor epistemology, which typically favours a social-and trauma-based understanding, intertwines such theories by conceiving self-harm as an embodied practice embedded in self-experience as related to others and one’s sociocultural sphere (Baker et al., 2013; Chandler, 2016; Donskoy and Stevens, 2013; Pembroke, 1996; Simopoulou and Chandler, 2020). As such thinking frames self-harm as existing within the social context, as opposed to solely being an individualistic behaviour, there is a clear need for its de-medicalisation (Chandler et al., 2011).

This analytical AE is within a survivor epistemological standpoint, exploring my LE of self-harm. It provides a critical overview, considering the felt experience of self-harm as situated within social context. This work is interdisciplinary, intertwining psychiatric and survivor knowledge, presenting both etic and emic perspectives that are negated (Chandler, 2016). I highlight suicidality within self-harm as significant, not to necessarily end one’s life, but to ‘kill off’ distressing experiences. I further position both self-cutting and eating distress (restrictive eating and excessive exercise) as self-harm, due to my experience of their phenomenological overlap. Likewise, many with LE may view eating distress, or self-cutting, as a continuation of self-harm but if not, will frequently present with both (Farber Klayman, 2002; Gosling et al., 2023; Gurung, 2018; Kettlewell, 1999; Kokaliari and Berzoff, 2008; Motz, 2009; Turp, 2003). Where necessary, I critically indicate potential differences or reiterate similarities.

## Methods

AE contextualises the researcher, their identity and positionality, within sociocultural context to elucidate phenomena (Boylorn and Orbe, 2014). AE shares ‘epistemological ground’ (Zhang, 2024: 186) with phenomenology in asking the researcher to meaningfully consider their experiential subjectivity in relation to others, considering ‘what happens to us, what we see with our own eyes, what we feel with our own bodies’ (Zhang, 2024: 186). Thus, AE as a phenomenological approach may produce knowledge that can more fully elucidate real-world experiences than conventional positivism (Mapp, 2008).

AE can be broadly compartmentalised into two types: one is evocative AE, which prioritises the person’s subjectivity (Adams et al., 2013). However, this approach is often criticised for narcissistic solipsism by offering insufficient sociocultural insights (Anderson, 2006). However, this critique risks epistemic injustice and violence by undermining the intrinsic value of LE perspectives in elucidating mental distress, which are often misunderstood (see Gunnarsson, 2021; O’Connell, 2023). Conversely, the analytic AE interweaves subjective experience with current evidence, seeking to establish greater applicability to sociocultural context, where the ‘I’ becomes more explicitly interspersed with the ‘We’ (Adams et al., 2013). This complements the survivor epistemological position for LE researchers seek “to move reflexively between a critical understanding of our own experiences, the points of intersection and departure with others” (Sweeney, 2016: 39), transitioning between the ‘I’ and ‘We’ to produce collective ways of understanding mental distress (Sweeney et al., 2009).

I undertook an analytical AE in the first term of my doctorate. I was motivated to reflect upon my LE because I have never been able to ‘recognise [myself] in the body of clinical research on self-harm’ (Donskoy and Stevens, 2013: 65). However, as my social/outsider identity is that of a clinical academic, I can share my LE perspective without restriction (Ellis et al., 2010). Therefore, I felt an evocative AE would not have been sufficiently inclusive, reflexive and self-accountable (Jackson and Mazzei, 2008). Conversely, an analytical AE may more effectively achieve such introspection, illustrating ‘facets of cultural experience, and (. . .) make characteristics of a culture familiar for insiders and outsiders’ (Ellis et al., 2010: 4).

I chronologically present anonymised vignettes, drawn from memory recall, my medical notes – from secondary mental health services – and diary entries written for personal reflection from 2011 to 2023. Event selection was guided by explorations of the felt meaning and LE of self-harm, its interplay with mental distress and relationships with self, others and wider social contexts. Each vignette was analysed comparatively to non-empirical first-person self-harm narratives and empirical studies conducted by researchers with and without LE (Pitard, 2017).

The underlying theoretical approach was social constructionism and within a survivor epistemological standpoint (Sweeney et al., 2009). Social constructionism understands that individual interpretation and subjectivity define and cognise reality and this is contextualised within temporal and sociocultural discourse (Andrews, 2012). This complements survivor epistemology which centralises the first-person perspective and, as mentioned, most commonly emphasises a social- and trauma-based understanding of mental distress (Sweeney et al., 2009; Sweeney and Beresford, 2020).

In this AE, I provide an overview of self-cutting as an adolescent, from 14 to 19 years old. I also explore a psychiatric understanding of self-harm as guided by professional experiences. I reflect upon my work supporting adults who have offended, often presenting with self-harm, in prison and then with adults with eating distress confined to a psychiatric inpatient unit. Further, I reflect upon my experiences of psychiatric services as a service-user. Finally, I reflect upon my LE of eating distress, from 26 to 27 years of age, in combination with prior experiences of self-cutting, to present a re-theorisation of self-harm. This first-person perspective emphasises self-harm as an embodied emotional practice mediated by relational and social conflict.

## Ethical considerations

Formal ethical approval was not required. However, AE incurs ethical quandaries as it may implicate non-consenting others (Adams et al., 2013). If possible, informed consent should be sought. However, if not, precautions must be taken, such as data anonymisation or alteration (Philaretou and Allen, 2006). Consequently, all vignettes are anonymised and events are detailed ambiguously, without alluding to particular people. This may exclude significant findings, but it is the narrative’s meaning, not specific details, that is deemed most significant.

Further, in drawing upon other survivor work, I risk proprietorially owning others’ stories to validate my own, homogenising knowledge and ‘repackaging’ experience, as psychiatric discourse does across research and clinical practice (Russo, 2016). I spoke with people with LE throughout, and reflected with supervisors, one of whom is a survivor researcher, to mitigate testimonial injustice. Such critical reflections with supervisors, alongside a reflexive journal, also mitigated the emotional labour associated with survivor research (Faulkner and Thompson, 2023).

## Reflexivity

Reflexivity allows for introspection and interpretation of researcher’s ideology, subjectivity experiences and identities within sociocultural context to make clear how such positionality influences research process and is central to AE (Boylorn and Orbe, 2014). Consequently, it was important to critically reflect upon how my positionality has influenced my interaction with mental health services and thus, explicitly present how my identities and perspectives of self-harm underpin this work.

‘Survivor identity does not itself represent a more ethical approach in research’ (Russo, 2016: 224) and sharing experiences is not always sufficient to disrupt dominant discourse. This is pertinent because AE still focuses on white people, centring a dominant viewpoint (Fixsen, 2023). Indeed, although I identify as a queer LE researcher, I am also a cisheteronormative presenting, middle-class, white academic and nurse, whose historic perspectives of mental ‘illness’ leant far more medicalised conceptualisations than they do now. Consequently, I had a fairly easy journey through mental health services. It could have been significantly different were I not white and had I been more outspoken, less ‘cooperative’, and thus perceived as more ‘difficult’, for I may have been less likely to receive support but more likely to be detained (NHS England Digital, 2021; Pembroke, 1996). Consequently, I often feel an imposter in peer spaces, knowing I also have been complicit in a system that oppresses distressed people, causing harm (Lawrence et al., 2022; Russo, 2016).

Concurrently, I do not feel like a nurse: clinicians refer to mental distress pejoratively to me, not realising my identity. It is as though I am too much of a nurse to be a service-user, and vice versa: I occupy a tangential space where one identity negates the other. Therefore, I initially avoided discussing professional experience, fearful of not presenting myself positively and delegitimising my survivor identity (Deliovsky, 2017). However, I realised that it was vital to remain honest for to repress essential aspects of the story would reinforce systemic discrimination and power differentials in psychiatry (Russo, 2016).

In psychiatric practice and research, self-harm is commonly constructed as an unhelpful behaviour requiring eradication. I do not recognise myself or my experiences within this discourse. Instead, in not seeing self-harm according to this clinical conceptualisation, I feel silenced, and in my failing to cease behaviourally, I am made deficient, ultimately substantiating past trauma. From my perspective, self-harm cannot be solely categorised as negative for, to me, it represents a multitude of meanings existing simultaneously: it is unhelpful and helpful, unwanted and wanted, one’s voice and silence, an active choice whilst being passive to something beyond one’s own understanding, self-violence and -care, to be in control and out of control and to be set apart from others whilst seeking a sense of belonging (Heney, 2020). Further, I cannot adhere to clinical priorities seeking to stop self-harm, for I argue that at best, this misunderstands its phenomenology, and at worst, risks perpetuating the silencing central to abuse and trauma, as they fail to recognise what self-harm represents. I seek for an alternative: to look to ourselves as healthcare professionals, critically considering our own complicity in perpetuating the oppression and discrimination of people with LE. Within this, there needs to be an acknowledgement of how we consistently fail to consider the person’s understanding of how their subjective experiences may mirror self-harm, including how it is proportionate to circumstance. Ultimately, we need to show that people with LE deserve, not censure, but to be heard and seen by the people who purport to care for them.

Overall, this AE embeds self-harm within survivor epistemology. However, it centres a white academic and nurse, substantiating contemporary self-harm research that already prioritises the young, White British woman excluding underrepresented, marginalised perspectives (Chandler et al., 2011; Gunnarsson, 2023). However, in undertaking this AE, I wish not to speak for others, but to create a dialogical, healing space that advocates for a more compassionate understanding of self-harm as reflective of and embedded within complex, intersecting sociocultural contexts, not individual deficiency.

## Becoming a self-harmer

This section considers self-harm from a young person’s lens from onset at 14–19 years old.

### The beginning of self-cutting

My initial self-harm engagement grew insidiously over time but was not gruesome, loud or impulsive (Chandler et al., 2011), but curious, private and self-sanitising (Chandler, 2016):

*I dug my fingernails into my arm out of frustration. I felt a release of something, and I found it useful for self-discipline and -improvement. Later, I used scissors. This would often involve a single cut, or multiple scratches, on my left arm or hand. This did not necessarily break the skin* (Memory recall).

Self-harm originated from complex social and relational interactions at school and home, which cemented my desire to be good, worthy and self-contained:

*I learned that people change, without any provocation, and their emotions differ from their behaviour. I felt hypervigilant to any lability. To cope, I sought to never acknowledge any anger, or anything else. I believed volatile emotion caused pain and fear. Eventually, I no longer understood what I was feeling. I saw this as self-preserving and empowering. However, I felt I was ‘too much’ for myself and for others* (Memory recall).

Self-cutting was therefore embroiled in wanting to hide inside myself, have self-control and protect other people. It was not possible to be authentically open, so I limited myself to being fully with other people. In my silence, ‘. . .I was a good girl (. . .) a quiet girl, no tantrums’ (Smith, 2006: 30), for self-harm gave me my voice, whilst maintaining my silence, removing ‘the need not only to talk about [feelings], but also to learn how to talk about them’ (Norman et al., 2023: 10157).

In expecting lability from others and fearing my emotional responses, I became sensitive to perceived rejection by romantic partners, with one episode described here:

*There was a cacophony of abandonment and rage so I could not see anything. I had shouting in my head, but I could not hear what was being said. Amid this, I had one rational voice, telling me not to cut, whilst another commanded I should. I heard CUT in my head and saw cuts on my left arm. I felt my fingers twitch from the inside, preparing. The culmination of this felt chaotic, a tangle of everything all at once. I did not have the words to say how I felt, nor did I know how to: they were trapped in my throat. After, I cried and tended to the wound compassionately, but felt ashamed, worried that he would leave because of my insanity. Later, I longed for him to see me but was silent. When he did, he dressed the wound* (Memory recall).

Contrary to clinical discourse which posits self-harm as an individualistic abnormality, self-harm exists relationally, for it represents a longing for meaningful connection, comfort and validation from others whilst expressing one’s fear for the preconceived loss that closeness entails (Chandler, 2016; Motz, 2009). This experience can be contextualised within historical trauma, such as childhood abuse, or relational conflict, wherein emotionality was delegitimised, invalidated, or ignored, so that people learnt their distress was insufficient or inappropriate (Gosling et al., 2023; Gurung, 2018; Morris et al., 2015). Clinical discourse often relies on abuse as an explanatory cause but frequently fails to adequately explore the subjective impact this has on a person, their relationship with themselves and others and how abusive experiences may be embedded in wider social inequity (Chandler et al., 2011).

Nevertheless, the person is considered abnormal and must learn ‘adaptive’ coping. I struggled with disclosure because of this, fearing I was inherently different, as cemented by homophobia:

*Being gay did not fit the narrative of what was around me. At school, many spoke about their disgust of LGBTQIA+. One girl told a teacher who identified as a lesbian that it ‘went against her religion’* (Memory recall).

Discrimination equating queerness to badness systematically undermines a person’s sense of themselves (Gosling et al., 2023). Self-harm soothes this, whilst paradoxically enforcing alienation: ‘I know that the more trapped I feel the more it helps. It makes me feel free. But then it traps me too’ (Baker et al., 2013, p15). Consequently, I downplayed my experiences, even to myself, stating self-harm occurred *‘very rarely actually’* and I hadn’t *‘done it for a good few weeks and have only been close to [it] a couple of times’*, so I was ‘*okay*’ (Diary excerpt, 29th of December 2011). Later, I more openly discussed my struggles with feeling abnormal: ‘*Maybe I am mad’* (Diary excerpt, 26th of October 2016), as consistent with others: ‘When I first cut up, I thought I was mad’ (Pembroke, 1996: 14; Smith, 2006). Speaking pejoratively about oneself may be then reinforced socially: ‘I just think she’s attention seeking (. . .) they wouldn’t be putting on a show (. . .) you’d do it and you’d keep it private’ (Klineberg et al., 2013: 7). I agreed: keeping self-harm private indicated self-reliance. This may arise from society’s propensity to value stoicism (Chandler, 2016).

Overall, the origin of self-harm may be socially and relationally oriented, with internalised feelings of difference compounded by sociocultural disapproval of both the self-harm and intersectional identities (Gosling et al., 2023). This reinforces self-harm by enhancing self-alienation and limiting disclosure to avoid being ‘othered’.

### The diffuse ‘inner’ world of self-harm

Clinically, self-harm is associated with alexithymia (an ‘inability’ to recognise and manage emotionality; Norman et al., 2023), wherein self-harm manages overwhelming emotions, constructed as distinct and separable (Klonsky et al., 2014). Such conclusions are binary, presupposing that everyone experiences emotionality the same (Norman et al., 2023). However, self-harm may not just be about ‘deal[ing] with feeling overwhelmed’ (Baker et al., 2013: 142) but an immersive cacophony:

*‘. . . when I (. . .) take a dip I start to panic and (. . .) I do not feel like myself at all I feel lost and like I am skating on really thin ice and at any moment I may fall through and drown (. . .) it feels like drowning. Like there is no beginning, middle or end (. . .) I feel like it is never going to end (. . .) my mind is a bit lost and there is no clarity to my thought-processes. It is like my brain contains lots of endless pieces of tangled up string – or perhaps (. . .) different coloured smoke twirling around one another – but there is no solidity to them’* (Diary excerpt, 7th of December 2016).

Similarly, others often describe feeling ‘a lot’ (Norman et al., 2023: 10155), or ‘so much at once’ (Stänicke, 2021: 9) and so ‘cut to quiet the cacophony’ (Kettlewell, 1999: 111). In common discourse, this experience may be solely described as ‘panic’ (Chandler, 2012). This risks oversimplification by assuming homogeneity across experiences whilst negating how emotionality is informed by wider social constructs of ‘acceptable’ versus ‘unacceptable’ emotion, that may amplify or lessen distress (Chandler, 2012; Gurung, 2018).

Nonetheless, my early examination of self-harm tallied with clinical interpretations, compartmentalising self-harm, but excluding significant detail:

*‘. . . when I am stressed or absolutely out of my head with anxiety, I scratch my arm*’ (Diary excerpt, 9th of November 2011).

Others may speak similarly: ‘I had begun to struggle with my emotions and how I felt. Feeling low was becoming a common feeling for me, along with some other emotions (anger, anxiety, confusion) (. . .) It helped me because (. . .) self-harming enabled me to feel (. . .) positive’ (Baker et al., 2013: 199). It might be that people sometimes rely on professional interpretations because self-harm feels incomprehensible (Klineberg et al., 2013). This is not to dismiss peer experiences, but to highlight that medicalised understanding permeates personal knowledge, explaining distress objectively so it makes sense to other people (Beresford et al., 2010; Sweeney et al., 2009). However, this may overlook social complexity. Further, such language may not empower the person to authentically feel self-harm (Baker et al., 2013), because ‘it’s one thing to have language, it’s quite another to have the right language (. . .) that speaks to the self, for the self, about the self’ (Baker et al., 2013: 98). Unfortunately, people are not supported to do this. Instead, the superficiality of distinct emotions is relied upon to prioritise, not one’s own understanding, but clinicians’, and others’.

Overall, instead of categorical ‘negative’ emotions guiding self-harm, there may be an amalgamation of immersive, impermeable psychic experience. This perspective sits counter-initiatively to clinical discourse, that describes emotions simplistically, negating complexity (Chandler, 2016).

### ‘Proper’ self-harm

For me, self-harm ensured the good supersedes the bad. This was cemented by education, as I channelled being ‘good’ into academic success:

*If I did not retain enough information during revision, I could dig my fingernails into myself as hard as possible and I would be better next time* (Memory recall).

Likewise, others feel self-harm aids productivity: ‘Every couple of hours, I would, just, make like one little cut and then I could (. . .) focus’ (Kokaliari and Berzoff, 2008: 265). Cutting seems to draw ‘a line in the sand’ (Kettlewell, 1999: 57), providing balance. However, my inability to ‘self-manage’ was a failure in and of itself, so I felt constantly on the verge of making mistakes:

*I sought to ‘punish myself’ and ‘when I did I felt like I was saying to myself, “This is because you are not good enough”’* (Diary excerpt, 26th of October 2016).

This prompted self-hatred: *‘I sometimes just hate myself more than anyone and I don’t know why’* (Diary excerpt, 21st of December 2011). Others describe similar experiences of being ‘bad’ as prompting a need to ‘dispel the toxic food inside’ (Motz, 2009: 147), or ‘[cut] the bad out’ (Pembroke, 1996: 23) . In seeking ‘goodness’, I sought to create deeper wounds:

*‘I have the faint trace of cuts. I’m even a failure at self-harm – can’t even make myself bleed’.* (Diary excerpt, 21st of December 2011); *‘Every time I do self-harm I think about the fact that I am not even very good at that – I never really bleed or scar. I want to scar’.* (Diary excerpt, 26th of October 2016).

This urge to ‘succeed’ at ‘proper’ self-harm may arise from sociocultural representation of the ‘typical self-harmer’ as causing permanent bodily damage (Chandler, 2016). It is also maintained in healthcare, whereby wounds are described as ‘superficial’ versus ‘severe’. Therefore, people with LE may underplay their distress, feeling discomforted in not deserving the ‘self-harm’ label (‘If I think of myself as a self-harmer, I’m making myself out to be worse than I am’) (Chandler, 2016: 41), Indeed, I felt more of a ‘self-harmer’ when I discovered razor use, around age 22:

*You can tear apart a razor and use that. This hurt less and was more effective because wounds scarred* (Memory recall).

This may also invoke doubting one’s authenticity, which was particularly apparent when I began to self-harm through eating distress later on: ‘*If I weigh “too” much then it would mean I wasn’t sick anymore and then I wouldn’t be deserving of help and treatment*’ (Diary excerpt, 1st of February 2023). Likewise, O’Connell (2023) desired ‘the diagnosis of anorexia itself’ because then she was ‘*really sick*’ (O’Connell, 2023: 270).

Cutting ‘properly’ – or likewise being a ‘proper anorexic’ – is more conventionally associated with a ‘self-harming identity’ in comparison to other methods (Chandler, 2016). This implicates people’s engagement, their self-concept and what self-harm is, a concept as yet hard to define.

## Self-harm within institutions

This section presents my experience of self-harm in institutions. This builds upon the last section, exploring negative clinical perceptions to consider how ‘severity’ and compartmentalisation of ‘psychopathological symptoms’ impacts clinical interpretation of distress and justification of restrictive practice.

### Professional interpretation of self-harm

Some with LE argue that self-harm is ‘a sane response’ when ‘people are gagged (. . .) yet are expected to behave in a controlled manner’ (Pembroke, 1996: 5). However, clinically, self-harm continues to be identified as an abnormal expression of *individualised* emotional distress, and the person with LE themselves becomes solely culpable for their ‘illness’ (Chandler et al., 2011). This was apparent on the psychiatric unit where pacing was identified as ‘anorexic’:

*We were encouraged to stop patients pacing but they were locked up, why would they not pace? Tigers in zoos pace* (Memory recall).

Self-harm is considered separate from the norm, reflecting a continuing dichotomisation: there are ‘these other people’ (Turp, 2003: 216), who cut and people who cope ‘appropriately’ (Chandler, 2016; Turp, 2003). However, whilst considered socially unacceptable, self-harm can be argued to be one of many self-destructive methods people use to self-soothe, such as binge-drinking or smoking (Turp, 2003). In this way, it can be considered as ‘part of ourselves (. . .) being acted out’ (Turp, 2003: 216).

However, such ‘symptomology’ is considered psychopathological and healthcare professionals categorise mental distress, and self-harm, according to severity (Baker et al., 2013; Farber Klayman, 2002; Turp, 2003). This classification of ‘superficial’ versus ‘severe’, as mentioned, enables clinical prioritisation, such as wound assessment for self-cutting and the use of body mass index to diagnose anorexia nervosa. This is despite ‘atypical anorexia’ occurring in ‘normal’-BMI also risking significant physical compromise (Vo and Golden, 2022). Further, this prioritisation may influence professional perceptions such that ‘superficial/mild’ equates to less authenticity, as in my self-harm experience, and substantiated here:

*In prison, ‘superficial’ self-harm became every-day. Although I tried to be supportive, colleagues and I felt as though our time was better spent treating people deemed more ‘serious’, for it was those tying ligatures and jumping off landings who might die* (Memory recall).

Within clinical practice, symptoms are eradicated through task-oriented approaches as preventing ‘problem’ behaviours achieves a ‘cure’ (Lawrence et al., 2022). One way in which this is apparent is through the cognitive-behavioural theorisation of eating ‘disorders’ (CBT-E). This compartmentalises psychopathological symptoms (‘abnormal’ eating patterns, low weight and compensatory mechanisms such as exercise) to eradicate, or the very least reduce, them (Muratore and Attia, 2021). On the psychiatric unit I worked on, this was apparent in the behavioural and chemical restrictions employed to eliminate exercise (Lawrence et al., 2022; Muratore and Attia, 2021):

*A patient was confined to her bedroom on a 1:1 to ensure she did not move around; a consultant repeatedly suggested a low dose of antipsychotics to reduce a patient’s pacing, despite her refusing many times* (Memory recall).

Other more implicit restrictive practice was utilised to prompt meal completion and thus ensure weight restoration. This included disapproving and frustrated attitudes and policing to ensure people with LE adhered to their ‘treatment’ (Komarova et al., 2024):

*Patients were instructed to finish meals. I asked a patient who did not to stay behind, like a student and a teacher, to ‘encourage’ her. Looking back, I undermined her, did not acknowledge her distress or commend her for trying* (Memory recall).

Coercion (or implicit restrictive practice) disrespects people with LE, negatively impacting their psychological wellbeing, losing sight of them as much more than their diagnosis, as whole and as human, reflected upon here (Lawrence et al., 2022):

*A patient, who was particularly thin, wanted desperately to be home. I perceived staff as not taking this seriously, perceiving it only as a desire to remain ‘anorexic’, rather than respecting her self-awareness that she was unlikely to improve in hospital. After many months of her saying the same thing and not improving, a community treatment order was eventually suggested* (Memory recall).

Enforcing bed rest and controlling meals under the ‘moral’ oversight of a ‘rational’ clinician have changed little since the 19th century (Malson, 1998). I would question its efficacy, arguing instead that attitudes of reductionistic compartmentalisation of ‘symptoms’, as in CBT-E, and associated use of restrictions, simply replicates the rigidity already apparent in eating distress, in which adherence to ‘correct rules’ solves the ‘problem’ (Lawrence et al., 2022). Luckily, the emphasis in my treatment was to (re)learn self-care by resting when needed. This taught me self-patience and how to be with myself, both of which I desperately needed. If pressured to adhere to strict dietary rules, I would have felt only a greater pull towards self-harm to regain my sense of self, safety and my control. This is reported by peers with LE: ‘Running represented an act of defiance (. . .), she ran as a life-sustaining practice that made recovery possible on her own terms’ (Schott and Langan, 2024: 9).

Overall, restrictive practice becomes justified because it is framed as in the patient’s ‘best interests’ and this means healthcare professionals can retain morality and good self-image. This is compounded by the litigious culture of health organisations, which seek to eradicate ‘risky’ behaviours that ultimately tarnish organisational representation. This is less about therapeutic support and more about justifying clinical decision-making (Pembroke, 1996). This attitude and the implementation of restriction practice becomes normalised (see Burrin et al., 2021), because it framed as necessary within clinical discourse, despite many with LE arguing that it misunderstands their distress, and is ineffective, punitive and abusive (Malson, 1998; Schott and Langan, 2024). Considering this, at what cost do we cause people long-lasting harm with the notion that ‘it’s for their own good’?

### Becoming a ‘mentally unwell patient’

My descent into becoming a ‘mentally unwell patient’ arose from witnessing recurrent violence and confinement of people with distress. However, my response felt unacceptable, being in sharp contrast to the normalisation of restriction in psychiatry and suicidal incidents in prison, so I suppressed it. However, I began to feel suicidal:

*I started to cut to practice suicide, wondering abstractly whether I would be brave enough to kill myself. I experimented with ligatures, to see what that felt like, and looked over bridges: ‘I feel trapped by myself – (. . .) I feel the most suicidal I have ever felt, really had a part of me egging me to [go] on to sit on a bridge (. . .) see what it felt like. . .’* (Diary excerpt, 5th of October 2020).

Self-cutting became a partial suicide (Donskoy and Stevens, 2013) to ‘kill off’ certain experiences, ‘sacrificing a part of [myself] to go on living’ (Le Breton, 2018: 39). Nestled within this was self-comfort, as I could rely on this when nothing else helped (Simopoulou and Chandler, 2020).

Upon entering secondary services, I sought to feel ‘worthy of support’ (Pembroke, 1996: 45), downplaying distress and avoiding emotional volatility to adhere to expectations of being ‘good’:

*I often ‘reiterated [my] “symptoms are not that bad” and that she is managing okay*’ (Excerpt from medical notes, 18th of January 2021).

I therefore ceased self-cutting, because self-harm is ‘bad’ and articulated my ‘symptoms’ in clinically palatable terms:

*I am reading about the role of alexithymia mediating self-harm (. . .) it has prompted me to think about my own difficulties in recognising and regulating my emotional state* (Diary excerpt, 15th of September 2020).

This meant I was taken seriously, whilst giving me a false sense of self-identification with ‘good’ insight. My trauma was referred to but never fully centred as most salient – I was diagnosed with *moderate to severe depression* (Excerpt from medical notes, 21st of December 2020) – and I therefore never learned authentically what such experiences meant for me, and how they would be always marked on my skin (Mate, 2022). However, I learned that I was an amalgamation of psychopathological symptoms that could be cured: I was ‘unwell’ and ‘different’, but I could be ‘better’.

## Towards a lived experience understanding of self-harm

This section explores a re-theorisation of self-harm. It is derived from my LE of self-cutting and eating distress as intertwined with LE narratives and trauma-informed, survivor, phenomenological, psychoanalytic and feminist approaches, presenting a direct critique of clinical discourse (Table 1).

**Table 1.** Psychiatric discourse versus AE narrative.

Psychiatric discourse	Autoethnographic narrative
Self-harm is an individually derived and maladaptive behaviour.	Self-harm is grounded in current and historical sociocultural and relational conflict. Self-harm can be identified as a reasonable attempt to negotiate with trauma, a means of surviving through difficult experiences.
Self-harm is indicative of arising psychopathological symptomology associated with mental illness.	Self-harm is a subjective, embodied practice and may tell of one’s story and experiences. These may include distressing and dismantling trauma and social distress.
Self-harm is a behaviour motivated by aversive negative emotionality, such as stress, anxiety, anger and sadness, and demonstrates abnormal personality traits, such as emotional dysregulation.	Self-harm incorporates a vast array of subjective embodied sensations, including tactile and sensate thoughts, feelings, and emotions that mediate, change and urge physical engagement. Such emotional experience is grounded in the person’s sociocultural context, including their intersectional identities and relationships with themselves and others.

### An overview of a lived experience understanding

**Figure 1.**
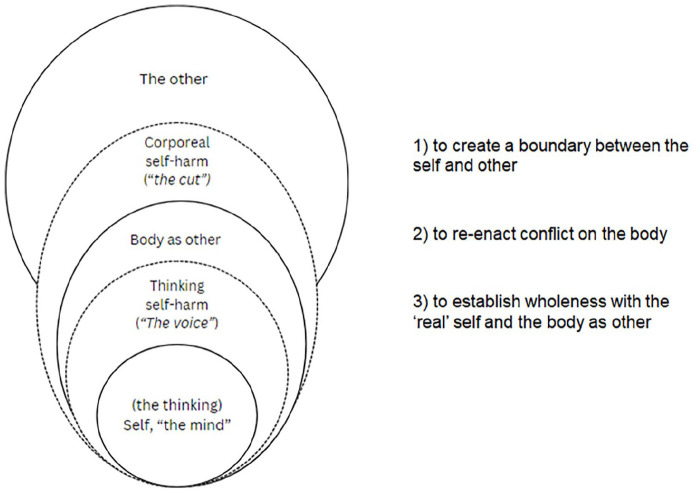
A lived experience perspective of self-harm.

(1) Immersive, aversive embodied emotionality, as representative of difficult experience, is located within the intersubjectivity of oneself/other (one’s body and other people), creating a psychic disembodiment. Self-harm may corporeally represent this divided conflict, (re-)integrating the self as whole whilst making visible sociocultural and relational experiences to oneself and others. In tangibly seeing oneself and one’s experiences on the skin, the person is enacting care that may not be given by others. However, whilst self-harm is self-protective, it reinforces inauthentic engagement due to individually driven shame and socially derived judgement. In positioning self-harm within social context, I emphasise that society inhabits each individual, and self-harm may retell the story of one’s experiences (Gunnarsson, 2022; Malson, 1998).
(2) To re-enact conflict through visibly making real one’s distress and inciting (self-)care and recognition from others and oneself (Baker et al., 2013; Motz, 2009; Simopoulou and Chandler, 2020)
(3) To create a boundary between the self and other, to protect against loss and vulnerability, whilst seeking meaningful connection (Motz, 2009) (see Figure 1).

### To be whole

In everyday life, the body fades into the background, existing as a coherent whole (Chandler, 2016; Motz, 2009). Alternatively, when distressed, one’s ‘inner’, thinking soul feels set apart from reality within a sensate prison. For me, this feels as though I am trapped behind ‘a wall’ (Supplemental Material A):

*‘There is such apparent disconnect from my body and thoughts, it is like being separated with several panes of glass, I cannot completely feel or engage with the world, myself or other people’* (Diary excerpt, 23rd of May 2023).

However, whilst absent in many ways, the body also feels overwhelmingly present with greater awareness of embodied experiences:

‘She described (. . .) the way the *images kept coming up and swirling round and round in her mind* (. . .) She *described a tight knot in her belly* (. . .) and she said her *skin felt hot and prickly*’ (Baker et al., 2013: 141, my emphasis).

This ‘tension’ (Kettlewell, 1999) exists in my stomach for eating distress and in my forearms and thighs for self-cutting (Supplemental Material B), as though ‘*I can feel my body and stomach’* (Diary excerpt, 1st of February 2023). Self-harm (re-)integrates this within oneself, providing balance: there is neither a before with negative emotion and an after without it, nor inner emotions and outer harm, but a ‘messy and indeterminate’ (Heney, 2020: 13) enmeshment of disparate experiences. Others describe this as a ‘release’ or ‘relief’ (Chandler, 2016): ‘When I burn myself and the pain goes up in an arch, there’s a wave, and, at the top, a moment when you can’t think or feel anything, like being blinded by sunlight’ (Barton-Breck and Heyman, 2012: 452).

Additionally, there may be reference to an ‘other’ that urges and visualises self-harm, in both cutting and eating distress: ‘*I felt a voice in my head suggest self-harm*’ (Excerpt from diary, 26th of November 2016). In AN, the ‘anorexic voice’ is often described as a ‘separate entity related to the self’ (Morrison et al., 2022: 1019), whereby there is a conflict between the ‘normal’ self and the other ‘evil twin’ (Morrison et al., 2022: 1029). I denote this is ‘the/my voice’, to capture it as being both internally generated whilst seemingly disparate. ‘The/my voice’ has pejoratively spoken to me in self-cutting and eating distress. It was not just a vocalised entity but embodied: it is as though the radio is turned up far too loudly, and it ‘*feels really big in my head’* (Diary excerpt, 20th October 2022). Whilst acknowledged as significant within LE discourse, self-harm ideation is under-emphasised in clinical research and practice, where the physicality is overtly focused on (Baker et al., 2013; Kettlewell, 1999).

Such embodied sensations may arise from difficult sociocultural and relational experiences that people internalise (Harris, 2000) as unwanted, aversive or unacceptable with oneself (Chandler, 2016; Motz, 2009). Others may tell the person they should not be feeling as they should, and so the person may then perceive such sensations, and themselves, as ‘shameful’, ‘dirty’, ‘bad’, ‘wrong’ or ‘evil’ (Baker et al., 2013). Self-harm can cleanse oneself of such putrefying experiences (Motz, 2009).

For instance, from childhood experiences, I concluded that all emotion, especially anger, must be ‘bad’. I therefore suppressed it behind ‘a wall’ for manageable containment, protecting and purifying myself. However, this suppression amplified the intensity of psychic experience, reinforcing beliefs I was ‘intense’ and intrinsically different from others, ultimately perpetuating self-harm. This hints at an early internalisation of gendered expectations for women and was substantiated later by eating distress. Indeed, I now reflect upon eating distress as an embodied mimicry of a previous relationship, a corporeal metaphor for what a ‘good girl’ in a heteronormative relationship should be: inferior, voiceless, and diminished. As such, I did not fear being fat but taking up any space at all (Malson, 1998), and sought to be disappear, because *with the starkness of my ribcage, my hip bones and striking jawline* (Memory recall), I was finally a ‘pure, clear shape’ (Fuchs, 2022: 113). Embedded within this experience was all-encompassing shame, for I had a ‘piercing awareness of [myself] as fundamentally deficient in some vital way’ (Mate, 2022: 30), validating a need to ‘cut out all the bad’ (Harris, 2000: 166). In this way, I was not a victim of psychopathology but enacting what I had learned I should be: silent and not ‘too much’. This also possibly represents wider patriarchal ideals for women: the desire for our erasure (Malson, 1998). The internalisation of this relational conflict informed ‘the/my voice’, like in others’ experiences (Morrison et al., 2022), mimicking comments from a previous partner:

*I was exercising but the/my voice was dogmatic: ‘You need to climb more. It’s not enough time spent, you aren’t doing it correctly’. I would confusedly ask it, ‘I am doing it?’ and ‘it’ answered, ‘not enough’* (Memory recall).

Eventually, by exercising intensely, ‘the/my voice’ would quieten, and I would be balanced. This was distressing and understandably, people with eating distress may externalise ‘the/my voice’ as a third person. This is considered important in treatment and recovery but represents the psychiatric tendency to reject a ‘pathological’ part of oneself that longs to be heard and is saying something important (Baker et al., 2013; Motz, 2009; Voswinkel et al., 2021).

Overall, difficult relational experiences induce aversive sensations that are labelled as abnormal, inducing a divided discomfort with one’s mind and body. Self-harm re-integrates the ‘bad’ to sustain the ‘good’, achieving wholeness with oneself.

### To care for oneself

Self-harm re-expresses previous trauma on the body to make distress visible to oneself and others. Simultaneously, it is an opportunity to care compassionately for oneself.

A person with self-harm may (re-)experience ongoing sensations to current conflict that replicate previous difficult experiences (Mate, 2022; van Der Kolk and Fisler, 1995). For experiences that live on in memory, self-harm is an embodied means of grounding distress in the present, making tangible that which is inexpressible, and needs to be (re-)experienced to make sense of it (Mate, 2022):

*Life goes on but if there is any implicit suggestion that a person may hurt themselves, or if I am not being heard, I could fall apart, and I feel every experience all at once, and I feel all that I am and was, and I cannot fully shake off the sense that I am not back before* (Memory recall).

The embodied representation of previous conflict also serves to legitimatise emotional experience physically, potentially to prompt visibility to others, but more importantly, to oneself (Chandler, 2016), to achieve what Steggals et al. (2024) notes as ‘intersubjective recognition’. For through self-harm you may see yourself when others cannot, or you otherwise cannot ask them or trust that they will:

*‘I almost want my insanity and pain visible on my body so that other people could see. . .’* (Diary excerpt, 26th of October 2016).

Self-harm also creates an opportunity for self-care, as the person can tend to oneself (Jackman et al., 2018: 9):

‘*I debated whether I needed stitches. I cried and tended to the wound compassionately, crying over and over, I’m sorry, I’m sorry’* (Memory recall).

This was replicated in eating distress, when in excessively exercising, I could rest freely.

Overall, self-harm is a bodily replication of conflict written on the skin and creates a space for self-resolution and -care. It is dynamic: both destructive and unwanted, whilst productive and soothing (Gurung, 2018; Motz, 2009; Simopoulou and Chandler, 2020; Turp, 2003), ‘repeated hurt, but also of repeated healing’ (Heney, 2020: 9), because ‘what were scars, after all, but wounds that had healed?’ (King, 2024: 476).

### To connect with others and oneself

Self-harm exists within the intersubjectivity, the shared, collective space, between one person and their body or another person (Motz, 2009; Turp, 2003; Supplemental Material C). It is a means of seeking meaningful connection with others and oneself.

I react intensely to vulnerability and perceived rejection, inherently distrusting others’ intentions (Motz, 2009). To cope, I ‘[retreat] into a numb and protective “shell”, as if there was a screen between [myself] and reality’ (Fuchs, 2022: 113):

*I created a wall to protect myself against other people and my experiences: I built a compartment for all embodied chaos, so that it was not me who was too much, but the space too small* (Memory recall).

In withdrawing inwards, self-harm risks isolation, but also incurs power, self-control and reliance by setting oneself apart from others. Concurrently, self-harm expresses that which is otherwise socially reprehensible, especially for women: self-assertion, -reliance, -‘violence’ and ‘excessive’ emotionality (Chandler, 2016). Subsequently, self-harm is paradoxically un-feminine (Malson, 1998): it gives us our voice, when we are not being seen and heard, and speaks for us. Simultaneously, self-harm may also symbolically maintain relational boundaries, for it prevents lashing out at others, whilst acknowledging one’s experience to oneself (Chandler, 2016; Motz, 2009). This may feel safer so that ‘rather than hurting the people I loved for hurting me (. . .) self-harm silenced the anger I felt’ (Baker et al., 2013: 10). However, I try to avoid self-harm now, not because it is ‘maladaptive’, or because I ‘should’ stop, but because I recognise it as detrimental to my relationship with myself, and other people:

*‘I am trying so hard to be authentic. I was reflecting (. . .) that I do not know if I have ever had a proper hug from another person. So much of my (adult) life has been me shying away from other people, that it is only recently where I feel perhaps I am being more honest with others and able to ask for their attention completely, without a wall’* (Diary excerpt, 31st of May 2023).

Consequently, self-harm attempts to reach out to others, and oneself, by expressing and acknowledging that which feels inexpressible, whilst preserving one’s own vulnerability, protecting relational boundaries.

## Conclusion

This re-theorisation of self-harm is grounded in survivor epistemology, contextualising my LE of self-harm with others’ and considering trauma-informed, feminist, psychoanalytic, sociological and phenomenological approaches. It conceives self-harm as a dynamic process of objectively contradictory but simultaneous meanings: of good and bad, destruction and productivity, isolation and connection, loss and gain, repression and expression. Overall, it is a corporeal manifestation of sociocultural and relational distress that engages with oneself and one’s experiences and substantiates what has happened, where other people cannot. This serves as a direct critique of positivistic concepts of mental distress that construct self-harm as an individualistic, psychopathological behaviour employed as a means of emotional dysregulation. By solely relying on psychiatric discourse, we judge people with self-harm needlessly, reinforcing their own beliefs that they are different, damaged and beyond help. If we were to instead seek to truly see the person, who they are and what has happened to them, we may contribute to healing.

## Supplemental Material

sj-docx-1-hea-10.1177_13634593251342902 – Supplemental material for ‘Why can’t you just be fine?’: An autoethnography of self-harm from a lived experience and nursing perspective Supplemental material, sj-docx-1-hea-10.1177_13634593251342902 for ‘Why can’t you just be fine?’: An autoethnography of self-harm from a lived experience and nursing perspective by Caroline da Cunha Lewin in Health
