# Supplementary material for: ‘Why can’t you just be fine?’: An autoethnography of self-harm from a lived experience and nursing perspective
Source: Health (London). 2025 May 26;30(2):193–213. doi: 10.1177/13634593251342902 (PMC12923617; doi:10.1177/13634593251342902)
Supplement: sj-docx-1-hea-10.1177_13634593251342902 – Supplemental material for ‘Why can’t you just be fine?’: An autoethnography of self-harm from a lived experience and nursing perspective [file sj-docx-1-hea-10.1177_13634593251342902.docx]

**Supplementary Materials**

**
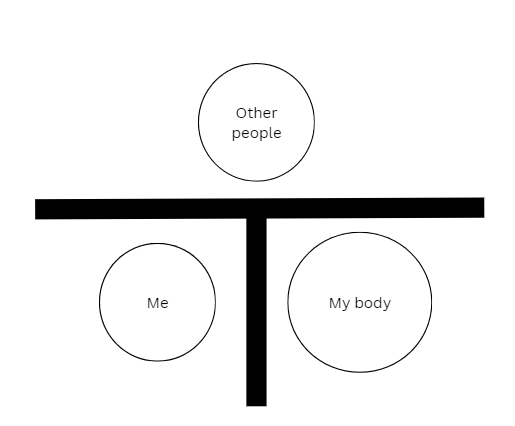
Supplementary Material A:** ‘The wall’: my experience of self/body split in self-harm.

**
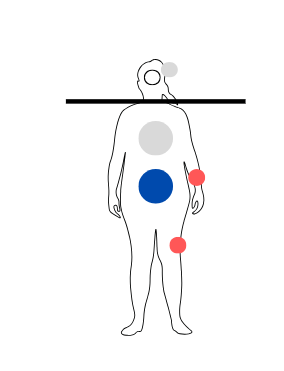
Supplementary Material B:** (Dis)embodiment in self-harm, a disconnect between mind/body as shown by the thick black line. Grey circles indicate the ‘static’ both in experience of ‘the voice’ and associated feelings in the chest; blue is the tension of eating distress in the stomach; red is the self-cutting in the forearm and thigh.

**
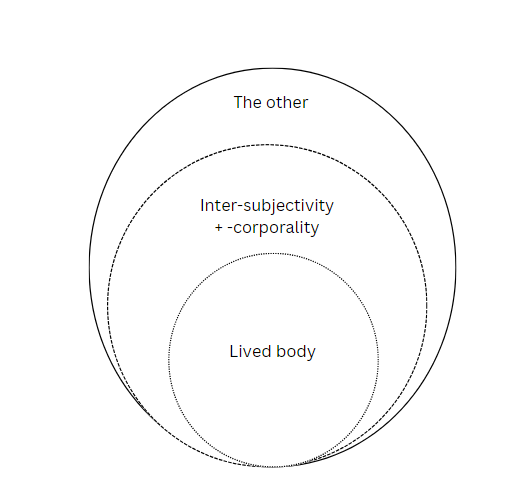
Supplementary Material C:** The shared space between self and other
